# Fair but Risky? Recycle Pricing Strategies in Closed-Loop Supply Chains

**DOI:** 10.3390/ijerph15122870

**Published:** 2018-12-14

**Authors:** Jianhong He, Lei Zhang, Xiao Fu, Fu-Sheng Tsai

**Affiliations:** 1 School of Economics and Management, Chongqing University of Posts and Telecommunications, Chongqing 400065, China; hejh@cqupt.edu.cn (J.H.); hjh69@sina.com (L.Z.); 2 Department of Business Administration, Cheng Shiu University, Kaohsiung 833, Taiwan; tsaifs@gcloud.csu.edu.tw; 3 Center for Environmental Toxin and Emerging-Contaminant Research, Cheng Shiu University, Kaohsiung 83347, Taiwan; 4 Super Micro Mass Research and Technology Center, Cheng Shiu University, Kaohsiung 83347, Taiwan

**Keywords:** closed-loop supply chain, irrational behavior education, fairness concerns, risk aversion, pricing strategy

## Abstract

We argue that a Nash bargaining model with behavioral factors (i.e., fairness concern and risk aversion) should be introduced to the price strategizing process in the context of a closed-loop supply chain. We consider three different pricing models: The first is when both the manufacturer and the retailer have fairness concerns; the second is when both the manufacturer and the retailer have risk aversion; and the final is when the manufacturer has risk aversion but the retailer has both risk aversion and fairness concern. Then we examine the model with game theory. The results have shown that fairness and risk aversion change the optimal pricing strategy, which affects the expected profits of retailers and manufacturers. The impacts of two (relatively irrational) behavioral factors on the wholesale and retail prices of new products, the recycle price and recycle transfer price of the waste products, are not the same. For new products, the wholesale price is most affected by behavioral factors and the sales price scores second. For waste recycling products, the transfer price is most affected by behavioral factors and the recycle price scores second. When considering fairness and risk aversion in retail, fairness concern is good for both manufacturers and retailers. This innovative pricing strategy model adds implications for sustainability in supply chain operations.

## 1. Introduction

A closed-loop supply chain(CLSC)refers to a supply chain system that incorporates reverse logistics and supports product recycling and life cycle management, which is more environment-friendly [1,2]. Whether the operation of the closed-loop supply chain is effective depends on the stakeholder’s focus on the interest game price, which has drawn much attention from researchers. As a pricing strategy of a closed-loop supply chain, studies have shown that the retailer recycling model is more effective than both manufacturer recycling and that of a third-party [3]. Emergent studies have focused on the differentiated pricing strategies of new products and remanufactured ones in closed-loop supply chains [4]. In such cases, factors including random demand and recovery, information asymmetry and the contractual sharing of supply risk are important to the price strategy and adjustment in the closed-loop supply chain [5,6]. In general, studies have contributed to issues of contract coordination mechanisms [7], channel mode choices [8], pricing strategies [9] and government interventions, assuming that decision makers will react absolutely rationally.

However, being absolutely rational is usually impossible for decision makers in closed-loop supply chain [10,11]. Hence, factors of individual behavior from social and cognitive psychology can be applied. For example, fairness concern is essential for decision makers’ profit distribution [12]. When fairness concern is applied, supply chain coordination can be achieved through wholesale price contracts [13]. The fact is, under these circumstances, problems such as coordination, optimal decision [14], equilibrium, and competition can all be solved [15]. Similarly, risk aversion is also largely applied in supply chain analysis. Decision makers tend to avoid possible risk, sometimes at the cost of profit [16]. Risk sensitivity also has a direct impact on optimal strategy. For example, manufacturers often apply different wholesale price strategies to deal with the risks associated with changing retailer attitudes toward risk [17]. Here, the more risk accepted by the decision makers, the less orders they place [18]. Different mechanisms should be set to coordinate the price strategy in closed-loop supply chains [19].

Existing studies often assume that there will be an irrational party on one side while the other side is absolutely rational [20,21]. In reality, however, both sides can be irrational in a closed-loop supply chain, leading to changes in the results of the pricing game [22,23,24]. Assuming that both sides be have irrationally, this paper attempts to study the price strategies of a closed-loop supply chain in the following three situations: First, both the manufacturers and retailers show fairness concern behaviors; second, both the manufacturers and retailers show risk aversion behaviors; third, manufacturers show risk aversion behaviors and retailers show both a concern for fairness and for risk aversion. Analyses have been conducted on how these two behavioral factors influence the optimal pricing and profit of members in a simulated closed-loop supply chain. The results provide reference for the management and decision making of closed-loop supply chains.

## 2. Descriptions and Assumptions

**Figure 1:**
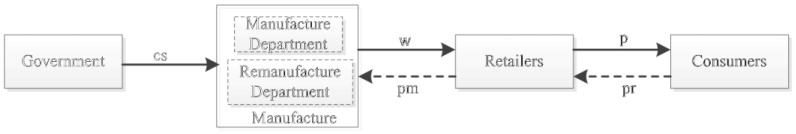
The closed-up supply chain under government subsides.

This paper focuses on the following: A closed-loop supply chain composed of a manufacturer and a retailer, where the manufacturer is responsible for the production of new and remanufactured products and the retailer retails products from the manufacturer and puts them into the market. The retailer is entrusted by the manufacturer to recycle waste at a set transfer price. Since manufacturers usually receive certain government subsidies for the disposal of waste, we consider government subsidies to be a fixed income that manufacturers obtain before decision making. Therefore, the closed-loop supply chain’ structure is as shown in Figure 1.

According to the description above, the assumptions and symbols of the model are as follows:

$c_{m}$ is the unit cost borne by a manufacturer when producing a new product from new raw material, a constant.

$c_{r}$ is the unit cost of remanufacturing recycled products, a constant. All recycled waste can be used, so the remanufacturing rate is 1 and $c_{m}>c_{r}$, $\Delta =c_{m}-c_{r}$.

p is the selling price of new products and remanufactured products and is the retailer’s decision variable, $p>c_{m}>c_{r}$.

w is the wholesale price of new products and remanufactured products sold by the manufacturer to the retailer, the manufacturer’s decision variable.

$p_{r}$ is the price of the waste when a retailer recycles it from the consumer, the retailer’s decision variable.

$p_{m}$ is the manufacturer’s transfer price, $p_{r}<p_{m}<c_{m}-c_{r}$ (to ensure that manufacturers and retailers can obtain profits and are motivated to do so), the manufacturer’s decision variable.

$c_{s}$ is the government subsidy factor for remanufactured products, the manufacturer’s decision variable.

D is the product demand. We suppose product demand D is a decreasing function of sales price p, that is, D(p)=α−βp. Among them, α is the potential demand of the market and β is the coefficient of price elasticity. We assume that the potential market demand is a random variable, $\alpha =\alpha_{0}+\varepsilon_{1}$, of which the expectation of uncertainty, $\varepsilon_{1}$, is 0 and the variance is $\delta_{1}{}^{2}$.

G is the supply of the waste market. We suppose the supply G is an increasing function of the recovery price $p_{r}$, $G\left(p_{r}\right)=a+bp_{r}$, of which a is the amount of waste that consumers would volunteer to give when the recycling price is 0 and b is the sensitivity of consumers to the price of recovery. The greater the value of b, the more sensitive consumers are. We suppose that a is also a random variable and that $a=a_{0}+\varepsilon_{2}$. The expectation of uncertainty, $\varepsilon_{2}$, is 0 and the variance is $\delta_{2}{}^{2}$.

When retailers show fairness concern behaviors, the manufacturer’s optimal wholesale price is $\bar{w^{fc^{*}}}$, the optimal recovery transfer price is $\bar{p_{m}^{fc^{*}}}$, the retailer’s optimal sales price is $\bar{p^{fc^{*}}}$, and the optimal return price is $\bar{p_{r}^{fc^{*}}}$.

When retailers show risk aversion behaviors, the manufacturer’s optimal wholesale price is $\bar{w^{ra^{*}}}$, the optimal return transfer price is $\bar{p_{m}^{ra^{*}}}$, the retailer’s optimal sales price is $\bar{p^{ra^{*}}}$, and the optimal return price is $\bar{p_{r}^{ra^{*}}}$.

Based on the assumptions above, the expected profits of manufacturers and retailers are:

(1) $$E\left(\pi_{M}\right)=\left(w-c_{m}\right)D+\left(c_{s}+\Delta -p_{m}\right)G$$

(2) $$E\left(\pi_{R}\right)=\left(p-w\right)D+\left(p_{m}-p_{r}\right)G$$

## 3. No Fairness Concern or Risk Aversion

Suppose both the manufacturer and the retailer are risk-neutral and fair-neutral in the closed-loop supply chain. That is to say, fair distribution of risk is taken and, in the closed-loop supply chain, is not a concern. In this case profit maximization is what drives decisions. Suppose the manufacturer is the leader of the market and the retailer is the follower. The Stackelberg game is as follows: First, the wholesale price, $w_{n}$, and the recycle price, $p_{m}$, of the new products are decided by the manufacturer based on demand. Then, the market price, p, and recycle price, $p_{r}$, are immediately set by the retailer.

Using the backward induction method, $\frac{\partial E\left(\pi_{R}\right)}{\partial p}=0$, $\frac{\partial E\left(\pi_{R}\right)}{\partial p_{r}}=0$, we get:

(3) $$\begin{array}{l} p=\frac{\alpha +w\beta }{2\beta }\\ p_{r}=\frac{bp_{m}-a}{2b} \end{array}\}$$

Equation (3) goes into $E\left(\pi_{M}\right)$ to get the first-order partial derivatives of w and $p_{m}$, let this be 0. With Equation (3) we obtain the optimal wholesale price and the transfer price of the manufacturer.

(4) $$\begin{array}{l} w^{*}=\frac{\alpha +c_{m}\beta }{2\beta }\\ p_{m}^{*}=\frac{\left(c_{s}+\Delta \right)b-a}{2b} \end{array}\}$$

Equation (4) goes into Equation (3).

(5) $$\begin{array}{l} p^{*}=\frac{3\alpha +c_{m}\beta }{4\beta }\\ p_{r}^{*}=\frac{\left(c_{s}+\Delta \right)b-3a}{4b} \end{array}\}$$

Equations (4) and (5) go into Equations (1) and (2) to obtain the expected profits of manufacturers and retailers.

$$E\left(\pi_{M}\right)=\frac{b{\left(\alpha -c_{m}\beta \right)}^{2}+\beta {\left[a+b\left(c_{s}+\Delta \right)\right]}^{2}}{8\beta b}$$

$$E\left(\pi_{R}\right)=\frac{b{\left(\alpha -c_{m}\beta \right)}^{2}+\beta {\left[a+b\left(c_{s}+\Delta \right)\right]}^{2}}{16\beta b}$$

**Proposition** **1.** *To achieve fair-neutral and risk-neutral optimal profit for manufacturers and retailers, there cycled price of the use products and the manufacturer’s transfer price will increase with the increase in government subsidies; meanwhile, the manufacturer’s wholesale price and the retailer’s sales prices are neither related to government subsidies nor to equity concerns or risk aversion.*

**Proof**  $$\frac{\partial w}{\partial c_{s}}=0,\;\frac{\partial p}{\partial c_{s}}=0$$

$$\frac{\partial p_{m}}{\partial c_{s}}=\frac{1}{2}>0,\;\frac{\partial p_{r}}{\partial c_{s}}=\frac{1}{4}>0,\;\frac{\partial E\left(\pi_{M}\right)}{\partial c_{s}}=\frac{1}{4}>0,\;\frac{\partial E\left(\pi_{R}\right)}{\partial c_{s}}=\frac{1}{8}>0$$

Proposition 1 shows that government subsidies for remanufacturing will help increase the recycle price, so as to increase the quantity and rate of use and profits to decision makers, which is beneficial to both manufacturers and retailers themselves.

## 4. When Fairness Concerns Exist for Both Manufacturers and Retailers

Equity concerns are usually demonstrated by the application of differences in earnings into the utility function. This can be shown as $U_{i}=\pi_{i}-\lambda_{i}\left(\pi_{k}-\pi_{i}\right)$, where $U_{i}$ is the member’s utility, $\pi_{i}$ is its own profit, $\pi_{k}$ is the other’s profits, $\lambda_{i}$ is the coefficient of fairness of interest, and $\lambda_{i}\geq 0$. When $\lambda_{i}=0$, the decision makers are fair and neutral; when $\pi_{i}\geq \pi_{k}$, their own utility increases with the increase in profit differences; and when $\pi_{i}<\pi_{k}$, the utility decreases with the increase in profit difference [25,26].

However, in reality, fairness is relative and both the power and contribution of decision making affect the fairness of income distribution. A feasible method is to construct a fairness-based framework based on the Nash bargaining game theory and, then, to further study the perception of fairness by both decision makers [27]. Based on this, the decision maker’s perception of fairness is assumed to be $\left(\tilde{\pi_{R}},\tilde{\pi_{M}}\right)$. The difference in benefits is supposed to result in changes in utility, that is, $U_{i}=\pi_{i}-\lambda_{i}\left(\pi_{k}-\pi_{i}\right)$. With the Nash bargaining game theory, we can assume a fair solution based on the following model:

(6) $$\begin{array}{l} \underset{\pi_{R},\pi_{M}}{\max}U_{R}U_{M}\\ s.t.\pi_{R}+\pi_{M}=\pi ,\tilde{\pi_{R}}+\tilde{\pi_{M}}=\pi \\ U_{R},U_{M}>0 \end{array}\}$$

Get

$$U_{R}U_{M}\left(\pi_{R}\pi_{M}\right)=\left[\left(1+\lambda_{r}\right)\pi_{R}-\lambda_{r}\tilde{\pi_{R}}\right]\times \left[\left(1+\lambda_{m}\right)\left(\pi -\pi_{R}\right)-\lambda_{m}\left(\pi -\tilde{\pi_{R}}\right)\right]$$

$U_{R}U_{M}\left(\pi_{R}\pi_{M}\right)\theta$ get the second order partial derivative $\frac{\partial^{2}\left(u_{R}u_{M}\right)}{\partial \pi_{R}{}^{2}}=-2\left(1+\lambda_{r}\right)\left(1+\lambda_{m}\right)<0$.

So, $U_{R}U_{M}\left(\pi_{R}\pi_{M}\right)$ is a strictly concave function. There is a unique maximum of $\tilde{\pi_{R}}$ and it is applicable in the following first-order conditions of

$$\frac{\partial U_{R}U_{M}\left(\tilde{\pi_{R}}\right)}{\partial \pi_{R}}=0$$

According to the fixed-point theorem, the fair solution of the Nash bargaining model can be obtained by:

$$\begin{array}{l} \tilde{\pi_{R}}=\frac{1+\lambda_{r}}{2+\lambda_{m}+\lambda_{r}}\pi \\ \tilde{\pi_{M}}=\frac{1+\lambda_{m}}{2+\lambda_{m}+\lambda_{r}}\pi \end{array}$$

The utility functions of manufacturers and retailers of Nash bargaining are:

(7) $$\begin{array}{l} U_{R}{}^{fc}=\frac{\left(1+\lambda_{m}\right)\left(2+\lambda_{r}\right)}{2+\lambda_{m}+\lambda_{r}}\pi_{R}-\frac{\lambda_{r}\left(1+\lambda_{r}\right)}{2+\lambda_{m}+\lambda_{r}}\pi_{M} \end{array}$$

(8) $$U_{M}{}^{fc}=\frac{\left(1+\lambda_{m}\right)\left(2+\lambda_{r}\right)}{2+\lambda_{m}+\lambda_{r}}\pi_{M}-\frac{\lambda_{m}\left(1+\lambda_{m}\right)}{2+\lambda_{m}+\lambda_{r}}\pi_{R}$$

The backwards induction model shows: $\begin{array}{l} \frac{\partial U_{R}{}^{fc}}{\partial p}=0,\frac{\partial U_{R}{}^{fc}}{\partial p_{r}}=0 \end{array}$

(9) $$\begin{array}{l} p^{fc}=\frac{\beta w\left(2+\lambda_{m}+\lambda_{r}\right)+\left(2+\lambda_{m}\right)\alpha -\beta c_{m}\lambda_{r}}{2\beta (2+\lambda_{m})}\\ p_{r}{}^{fc}=\frac{p_{m}b\left(2+\lambda_{m}+\lambda_{r}\right)-\left(2+\lambda_{m}\right)a-b\left(c_{s}+\Delta \right)\lambda_{r}}{2b(2+\lambda_{m})} \end{array}\}$$

By putting Equation (9) into Equation (8) we achieve the first-order partial derivatives of w and $p_{m}$ respectively. Supposing it is 0, we get the optimal wholesale price and the recycle transfer price of the manufacturer.

(10) $$\begin{array}{l} w^{fc}{}^{*}=\frac{\alpha {\left(2+\lambda_{m}\right)}^{2}+\beta c_{m}\left[4\left(1+\lambda_{r}\right)+\lambda_{m}\left(2+\lambda_{r}\right)\right]}{\beta \left(4+\lambda_{m}\right)\left(2+\lambda_{m}+\lambda_{r}\right)}\\ p_{m}{{}^{fc}}^{*}=\frac{-a{\left(2+\lambda_{m}\right)}^{2}+bc_{m}\left[4\left(1+\lambda_{r}\right)+\lambda_{m}\left(2+\lambda_{r}\right)\right]}{b\left(4+\lambda_{m}\right)\left(2+\lambda_{m}+\lambda_{r}\right)} \end{array}\}$$

Equation (10) goes into Equation (9) as follows:

(11) $$\begin{array}{l} p^{fc}{}^{*}=\frac{\alpha \left(3+\lambda_{m}\right)+\beta c_{m}}{\beta \left(4+\lambda_{m}\right)}\\ p_{m}{{}^{fc}}^{*}=\frac{-a\left(3+\lambda_{m}\right)+b(c_{s}+\Delta )}{b\left(4+\lambda_{m}\right)} \end{array}\}$$

Equations (10) and (11) go into Equations (1) and (2) to give the expected profit of both sides.

$$E\left(\pi_{M}^{fc^{*}}\right)=\frac{b{\left(\alpha -\beta c_{m}\right)}^{2}{\left(2+\lambda_{m}\right)}^{2}+\beta {\left[a+b\left(c_{s}+\Delta \right)\right]}^{2}{\left(2+\lambda_{m}\right)}^{2}}{\beta b{\left(4+\lambda_{m}\right)}^{2}\left(2+\lambda_{m}+\lambda_{r}\right)}$$

$$E\left(\pi_{R}^{fc^{*}}\right)=\frac{b{\left(\alpha -\beta c_{m}\right)}^{2}\left(2+\lambda_{m}+3\lambda_{r}+\lambda_{m}\lambda_{r}\right)+\beta {\left[a+b\left(c_{s}+\Delta \right)\right]}^{2}\left(2+\lambda_{m}+3\lambda_{r}+\lambda_{m}\lambda_{r}\right)}{\beta b{\left(4+\lambda_{m}\right)}^{2}\left(2+\lambda_{m}+\lambda_{r}\right)}$$

**Proposition** **2.** *When both the manufacturer and the retailer have fairness concerns the manufacturer’s optimal wholesale price decreases as the retailer’s fairness concern coefficient increases. This increases with its own fairness concern coefficient. The manufacture’s recycle and transfer price increases with the increase in the retailer’s fairness concern coefficient and decreases with its own fairness concern coefficient [28].*

**Proof**  $$\frac{\partial w^{fc^{*}}}{\partial \lambda_{m}}=\frac{\left(\alpha -\beta c_{m}\right)\left(2+\lambda_{m}\right)\left[4+6\lambda_{r}+\lambda_{m}\left(2+\lambda_{r}\right)\right]}{\beta {\left(4+\lambda_{m}\right)}^{2}{\left(2+\lambda_{m}+\lambda_{r}\right)}^{2}}>0$$

$$\frac{\partial w^{fc^{*}}}{\partial \lambda r}=\frac{\left(\beta c_{m}-\alpha \right){\left(2+\lambda_{m}\right)}^{2}}{\beta \left(4+\lambda_{m}\right){\left(2+\lambda_{m}+\lambda_{r}\right)}^{2}}<0$$

$$\frac{\partial p_{m}{}^{fc^{*}}}{\partial \lambda_{m}}=-\frac{\left[a+b\left(c_{s}+\Delta \right)\right]\left(2+\lambda_{m}\right)\left[4+6\lambda_{r}+\lambda_{m}\left(2+\lambda_{r}\right)\right]}{b{\left(4+\lambda_{m}\right)}^{2}{\left(2+\lambda_{m}+\lambda_{r}\right)}^{2}}<0$$

$$\frac{\partial p_{m}{}^{fc^{*}}}{\partial \lambda r}=\frac{\left[a+b\left(c_{s}+\Delta \right)\right]{\left(2+\lambda_{m}\right)}^{2}}{b\left(4+\lambda_{m}\right){\left(2+\lambda_{m}+\lambda_{r}\right)}^{2}}>0$$

Proposition 2 shows that with the increase in the manufacturer’s fairness concern coefficient, the effect of the retailer’s profit on the manufacturer’s utility is greater. In response, the manufacturer’s pricing decision is more aggressive [29]. This reduces the retailer’s bargaining power. At this time, the manufacturer increases the wholesale price of new products and reduces the transfer price of the waste to increase the proportion of profits in the supply chain. However, this inevitably causes the retailer’s fairness concerns. Then, with the increase in the retailer’s fairness concern coefficient, the manufacturer’s bargaining power is decreased so the manufacturers tend toward conservative pricing by choosing lower wholesale prices and higher waste recycling prices. Thereby the manufacturer’s proportion of profits in the supply chain is reduced.

**Proposition** **3.** *When both the manufacturer and the retailer have fairness concerns, the optimal sales price of the retailer increases with the increase in the manufacturer’s fairness concern coefficient; the optimal recycling price decreases with the increase in the manufacturer’s fairness concern coefficient. The retailer’s sales price and recycle price are not be affected by their own fairness concern coefficient, that is to say, whether or not there are fairness concerns, retailers will try to improve their own profits by adjusting the sale and transfer prices.*

**Proof**  $$\frac{\partial p^{fc^{*}}}{\partial \lambda_{m}}=\frac{\alpha -\beta c_{m}}{\beta {\left(4+\lambda_{m}\right)}^{2}}>0$$

$$\frac{\partial p_{r}{}^{fc^{*}}}{\partial \lambda_{m}}=-\frac{a+b\left(c_{s}+\Delta \right)}{b{\left(4+\lambda_{m}\right)}^{2}}<0$$

$$\frac{\partial p^{fc^{*}}}{\partial \lambda_{r}}=0,\frac{\partial p_{r}}{\partial \lambda_{r}}=0$$

Proposition 3 shows when both the retailer and the manufacturer show fairness concern behaviors. As market leaders, the stronger the manufacturer’s concern for fairness, the more likely it is for the manufacturer to increase the wholesale price and to decrease the recycle and transfer prices which in turn increases their proportion of profits in the supply chain. As a result, the retailer’s profits are reduced. As a response, the retailer raises sale prices and lowers recycle prices to pass the risk on to consumers and increase their own profits and the proportion of profits in the supply chain.

**Corollary** **1.** *(1)*$w^{fc^{*}}>\bar{w^{fc^{*}}},w^{*}>\bar{w^{fc^{*}}}$*;(2)*$p_{m}^{fc^{*}}<\bar{p_{m}^{fc^{*}}},p_{m}^{*}<\bar{p_{m}^{fc^{*}}}$*;(3)*$p^{fc^{*}}>\bar{p^{fc^{*}}}=p^{*},p_{r}^{fc^{*}}<\bar{p_{r}^{fc^{*}}}<p_{r}^{*}$*;(4)*$Q^{fc^{*}}<Q^{*},G^{fc^{*}}<G^{*}$.

Proof that only when retailers have fairness concerns, $\lambda_{m}=0$, $w^{fc^{*}}=\frac{\alpha +\beta c_{m}\left(1+\lambda_{r}\right)}{\beta \left(2+\lambda_{r}\right)}=\bar{w^{fc^{*}}}$, do we can see $w^{fc^{*}}$ as an increasing function of $\lambda_{m}$, so $w^{fc^{*}}>\bar{w^{fc^{*}}}\left(0<\lambda_{m}<1\right)$. Similarly, we know that $p_{m}^{fc^{*}}<\bar{p_{m}^{fc^{*}}}$, $p^{fc^{*}}>\bar{p^{fc^{*}}}$, and $p_{r}^{fc^{*}}<\bar{p_{r}^{fc^{*}}}$.

Corollary 1 shows that only when the manufacturer has fairness concerns should the optimal wholesale price of manufacturers be under that of the rational optimality. It also shows that when both sides have fairness concerns the optimal recycle and transfer prices should be higher than that of the rational optimality. This is because the bargaining power of manufacturers is starkly weakened only when retailers show fairness concern behaviors, so manufacturers tend to reduce the wholesale prices and raise the recycle and transfer prices, which increases the retailer’s profits. When both retailers and manufacturers have fairness concerns, the retailer’s optimal sales price is higher while the retailer’s optimal recycle price is lower than that of rational optimality or that of when only retailers have fairness concerns. Sales volume is a decreasing function of sales price while recycle volume is an increasing function of recycle price. When both retailers and manufacturers show fairness concern behaviors, the optimal sales volume and the optimal recycle volume will both be less than that of rational optimality.

## 5. When Both Manufacturers and Retailers Have Risk Aversion

Risk aversion refers to the decision of a company to avoid risks and possible losses [24]. As shown by many studies [27], the objective utility function that is applied to characterize risk aversion behavior is:

$$U^{aversion}\left(\pi \right)=E\left(\pi \right)-\eta_{i}\sqrt{Var\left(\pi \right)}$$

where $\eta_{i}$ is the risk aversion of decision makers. When $\eta_{i}>0$ the policy-makers are risk-averse and, when $\eta_{i}=0$ or when the expected utility of the policy maker equals the expected profit, they are risk-neutral.

Based on the research, the expected utility function for retailers and manufacturers is:

(12) $$U_{R}{}^{ra}=\left(p-w\right)\left(\alpha -\beta p\right)+\left(p_{m}-p_{r}\right)\left(a+bp_{r}\right)-\eta_{r}\left[\left(p-w\right)\delta_{1}+\left(p_{m}-p_{r}\right)\delta_{2}\right]$$

(13) $$U_{M}{}^{ra}=\left(w-c_{m}\right)\left(\alpha -\beta p\right)+\left(c_{s}+\Delta -p_{m}\right)\left(a+bp_{r}\right)-\eta_{m}\left[\left(w-c_{m}\right)\delta_{1}+\left(c_{s}+\Delta -p_{m}\right)\delta_{2}\right]$$

According to backwards induction, Equation (13) carries out the first-order derivation of p and $p_{r}$ respectively, $\begin{array}{l} \frac{\partial U_{R}{}^{ra}}{\partial p}=0,\frac{\partial U_{R}{}^{ra}}{\partial p_{r}}=0 \end{array}$.

(14) $$\begin{array}{l} p^{ra}=\frac{\alpha +w\beta -\eta_{r}\delta_{1}}{2\beta }\\ p_{r}{}^{ra}=\frac{bp_{m}-a+\eta_{r}\delta_{2}}{2b} \end{array}\}$$

When Equation (14) is put into Equation (13) we get the first-order partial derivatives of w and $p_{m}$, respectively, then, supposing it is 0, Equation (14) gives us the optimal wholesale price and the transfer price of the manufacturer:

(15) $$\begin{array}{l} w^{ra^{*}}=\frac{\alpha +c_{m}\beta +\eta_{r}\delta_{1}-2\eta_{m}\delta_{1}}{2\beta }\\ p_{m}^{ra^{*}}=\frac{-a+b\left(c_{s}+\Delta \right)-\eta_{r}\delta_{2}+2\eta_{m}\delta_{2}}{2b} \end{array}\}$$

When Equation (15) is put into Equation (14):

(16) $$\begin{array}{l} \begin{array}{l} p^{ra^{*}}=\frac{3\alpha +c_{m}+\beta -\eta_{r}\delta_{1}+2\eta_{m}\delta_{1}}{4\beta }\\ p_{r}^{ra^{*}}=\frac{-3a+b\left(c_{s}+\Delta \right)+\eta_{r}\delta_{2}-2\eta_{m}\delta_{2}}{4b} \end{array}\} \end{array}$$

$$D^{ra^{*}}=\frac{\alpha -c_{m}\beta +\eta_{r}\delta_{1}+2\eta_{m}\delta_{1}}{4}$$

$$G^{ra^{*}}=\frac{a+b\left(c_{s}+\Delta \right)+\eta_{r}\delta_{2}+2\eta_{m}\delta_{2}}{4}$$

The optimal sales volume is: $Q^{ra^{*}}=\frac{\alpha -\beta c_{m}+\eta_{r}\delta_{1}+2\eta_{m}\delta_{1}}{4}$.

The optimal recycle volume is: $G^{ra^{*}}=\frac{a+b\left(c_{s}+\Delta \right)+\eta_{r}\delta_{2}+2\eta_{m}\delta_{2}}{4}$.

When Equations (16) and (15) are put into Equations (1) and (2) we get the expected profits of manufacturers and retailers as well as that of the whole supply chain:

$$E\left(\pi_{M}^{ra^{*}}\right)=\frac{b\left\{{\left[\left(\alpha -\beta c_{m}\right)+\eta_{r}\delta_{1}\right]}^{2}-4{\left(\eta_{m}\delta_{1}\right)}^{2}\right\}+\beta \left\{{\left[a+b\left(c_{s}+\Delta \right)+\eta_{r}\delta_{2}\right]}^{2}-4{\left(\eta_{m}\delta_{2}\right)}^{2}\right\}}{8\beta b}$$

$$E\left(\pi_{R}^{ra^{*}}\right)=\frac{\begin{array}{l} b\left\{{\left[\left(\alpha -\beta c_{m}\right)+2\eta_{m}\delta_{1}\right]}^{2}-3{\left(\eta_{r}\delta_{1}\right)}^{2}-2\eta_{r}\delta_{1}\left[\left(\alpha -\beta c_{m}\right)+2\eta_{m}\delta_{1}\right]\right\}+\\ \beta \left\{{\left[a+b\left(c_{s}+\Delta \right)+2\eta_{m}\delta_{2}\right]}^{2}-3{\left(\eta_{r}\delta_{2}\right)}^{2}-2\eta_{r}\delta_{2}\left[a+b\left(c_{s}+\Delta \right)+2\eta_{m}\delta_{2}\right]\right\} \end{array}}{16\beta b}$$

**Proposition** **4.** *When both the manufacturer and the retailer show risk aversion behaviors, the manufacturer’s optimal wholesale price decreases with the increase in the manufacturer’s risk aversion coefficient*$\eta_{m}$*and the market potential demand variance*$\delta_{1}$*. Meanwhile, with an increase in the retailer’s risk aversion coefficient*$\eta_{r}$*and the market potential demand variance*$\delta_{1}$*the manufacturer’s optimal recycle and transfer price increases with the increase in the manufacturer’s risk aversion coefficient*$\eta_{m}$*and the market potential demand variance*$\delta_{2}$*, along with an increase in the retailer’s risk aversion coefficient*$\eta_{r}$*and the market potential demand variance*$\delta_{2}$.

**Proof**  $$\frac{\partial w^{ra^{*}}}{\partial \eta_{m}\delta_{1}}=-\frac{1}{\beta }<0,\frac{\partial w^{ra^{*}}}{\partial \eta_{r}\delta_{1}}=\frac{1}{2\beta }>0$$

$$\frac{\partial p_{m}{}^{ra^{*}}}{\partial \eta_{m}\delta_{2}}=\frac{1}{b}>0,\frac{\partial p_{m}^{ra^{*}}}{\partial \eta_{r}\delta_{2}}=-\frac{1}{2b}<0$$

Proposition 4 shows that with the increase in risk aversion, as the market leader, on the one hand, the manufacturer reduces the wholesale price to the lower the retailer’s sale price and increases the sales volume. In this way, the risk is transferred to the retailers. On the other hand, the manufacturer increases the recycle and transfer prices to encourage retailers to provide more used products to improve the recycle and remanufacturing volume, through which the manufacturer can increase profits and make up for part of the positive market loss. At the same time, with the increase in there retailer’s risk aversion, manufacturers take the opportunity to increase the wholesale price and reduce the recycle and transfer prices for more profits and a higher proportion of profits in the supply chain.

**Proposition** **5.** *When both the manufacturers and the retailer have risk aversion, the optimal sales price of the retailer decreases with the increase in the retailer’s risk aversion coefficient*$\eta_{r}$*and the potential demand variance*$\delta_{1}$*. It also decreases with the increase in the manufacturer’s risk aversion coefficient*$\eta_{m}$*and the potential demand variance*$\delta_{1}$*. The optimal recycle price of the retailer increases with the increase in the manufacturer’s risk aversion coefficient*$\eta_{m}$*and the potential demand of the market for the waste variance*$\delta_{2}$*. It increases with the increase in the retailer’s risk aversion coefficient*$\eta_{r}$*and the potential demand of the market for the waste variance*$\delta_{2}$.

**Proof**  $$\frac{\partial p^{ra^{*}}}{\partial \eta_{r}\delta_{1}}=-\frac{1}{4\beta }<0,\frac{\partial p^{ra^{*}}}{\partial \eta_{m}\delta_{1}}=-\frac{1}{2\beta }<0$$

$$\frac{\partial p_{r}{}^{ra^{*}}}{\partial \eta_{m}\delta_{2}}=\frac{1}{2b}>0,\frac{\partial p_{r}^{ra^{*}}}{\partial \eta_{r}\delta_{2}}=\frac{1}{4b}>0$$

Proposition 5 shows that as retailers increase their risk aversion, they, on the one hand, reduce the positive returns by reducing the sales price of new products, encouraging consumers to buy more new products and increasing the sales of new products. On the other hand, the more afraid retailers are of risk, for their own benefit, the higher the price of recycling waste products from consumers, increasing the recovery of waste products. The risk aversion of the manufacturer increases when the manufacturer, according to personal interest, raises the price of recycling and transfer so that the retailer may also raise their recovery price and obtain more benefits from the recycling of waste products.

**Corollary** **2.** *(1)* $p^{ra^{*}}<\bar{p^{ra^{*}}}<p^{*}$ *;* *(2)* $p_{r}^{ra^{*}}>\bar{p_{r}^{ra^{*}}}>p_{r}^{*}$ *;* *(3)* $w^{ra^{*}}<\bar{w^{ra^{*}}},w^{*}<\bar{w^{ra^{*}}}$ *;* *(4)* $p_{m}^{ra^{*}}>\bar{p_{m}^{ra^{*}}},p_{m}^{*}>\bar{p_{m}^{ra^{*}}}$

Prove that when only retailers show risk aversion behaviors, that is $\eta_{m}=0$, then the wholesale price of the manufacturer is $w^{ra^{*}}=\frac{\alpha +c_{m}\beta +\eta_{r}\delta_{1}}{2\beta }=\bar{w^{ra^{*}}}$. We know from Proposition 6 that $w^{ra^{*}}$ is a decreasing function of $\eta_{m}$, $w^{ra^{*}}<\bar{w^{ra^{*}}}$ ($0<\eta_{m}<1$ ) and that $\bar{w^{ra^{*}}}$ is an increasing function of $\eta_{r}$, so $w^{*}<\bar{w^{ra^{*}}}$. Similarly, $p^{ra^{*}}<\bar{p^{ra^{*}}}<p^{*}$, $p_{r}^{ra^{*}}>\bar{p_{r}^{ra^{*}}}>p_{r}^{*}$, and $p_{m}^{ra^{*}}>\bar{p_{m}^{ra^{*}}},p_{m}^{*}>\bar{p_{m}^{ra^{*}}}$.

Corollary 2 shows that when sides have risk aversion, the retailer’s optimal sales price is lower than the optimal sales price only when retailers have risk aversion. The optimal recycle price is higher than the optimal one only when retailers have risk aversion. This is because with the increase in risk aversion of both sides, retailers, in order to protect their own interests, will further reduce the sales prices to promote selling and increase the recycle price to obtain more profits. When it is only retailers with risk aversion, the manufacturer’s optimal wholesale price is higher than that of when both sides have risk aversion and are the rational optimal one. This is while the recycle price is lower and because when only the retailer has risk aversion the manufacturers increase the wholesale price to control its price-cutting and to increase their wholesale price while reducing the recycle price for more profits.

**Proposition** **6.** *(1)*$p^{fc^{*}}>p^{*}>p^{ra^{*}}$*;(2)*$p_{r}^{fc^{*}}<p_{r}^{*}<p_{r}^{ra^{*}}$*;(3)*$Q^{fc^{*}}<Q<Q^{ra^{*}}$*;(4)*$G^{fc^{*}}<G<G^{ra^{*}}$.

**Proof**  $$(1)\;p^{*}=\frac{3\alpha +c_{m}\beta }{4\beta }$$

$$p^{fc^{*}}-p^{*}=\frac{\left(\alpha -\beta c_{m}\right)\lambda_{m}}{4\beta \left(4+\lambda_{m}\right)}>0,\;p^{ra^{*}}-p^{*}=-\frac{\eta_{r}\delta_{1}+2\eta_{m}\delta_{1}}{4\beta }<0,\;\mathrm{so}p^{fc^{*}}>p^{*}>p^{ra^{*}}$$

$$(2)\;p_{r}^{*}=\frac{\left(c_{s}+\Delta \right)b-3a}{4b}$$

$$p_{r}^{fc^{*}}-p_{r}^{*}=-\frac{\left[a+b\left(c_{s}+\Delta \right)\right]\lambda_{m}}{4b\left(4+\lambda_{m}\right)}<0,\;p_{r}^{ra^{*}}-p_{r}^{*}=\frac{\eta_{r}\delta_{2}+2\eta_{m}\delta_{2}}{4b}>0,\;\mathrm{so}\;p_{r}^{fc^{*}}<p_{r}^{*}<p_{r}^{ra^{*}}$$

$$(3)\;Q^{*}=\frac{\alpha -\beta c_{m}}{4}$$

$$Q^{fc^{*}}-Q^{*}=\frac{\lambda_{m}\left(\beta c_{m}-\alpha \right)}{4+\lambda_{m}}<0,\;Q^{ra^{*}}-Q^{*}=\frac{\eta_{r}\delta_{1}+2\eta_{m}\delta_{1}}{4\beta }>0,\;\mathrm{so}Q^{fc^{*}}<Q<Q^{ra^{*}}$$

$$(4)\;G^{*}=\frac{a+b\left(c_{s}+\Delta \right)}{4}$$

$$G^{fc^{*}}-G^{*}=-\frac{\lambda_{m}\left[a+b\left(c_{s}+\Delta \right)\right]}{4+\lambda_{m}}<0,\;G^{ra^{*}}-G^{*}=\frac{\eta_{r}\delta_{2}+2\eta_{m}\delta_{2}}{4}>0,\;\mathrm{so}G^{fc^{*}}<G<G^{ra^{*}}$$

Proposition 6 shows that fairness concerns and risk aversion change the pricing strategies of manufacturers and retailers. When both sides show fair and responsible behavior, they play a positive role in the retailer’s sales price and have a negative effect on the recycle price. In order to obtain greater benefits, the rationality is maximized. However, when both sides have risk aversion, they both play a negative role in the pricing of the retailer’s sales and at the same time play a positive role in the recycle price. Therefore, based on the rational optimal, to improve the sales price and the optimal recycle price, and because sales volume is a decreasing function of sales price, the recycle volume is an increasing function of the recycle price, so the optimal sales volume and the optimal recycle volume under the risk aversion of both sides will be the largest.

## 6. Manufacturers Have Risk Aversion and Retailers Have Fairness Concerns and Risk Aversion

The equity concerns function and loss avoidance function for retailers can be shown as $E\left(\pi_{R}\right)\pm$ with additional effects. Similarly, the total expected utility function when retailers have fairness concerns and risk aversion can be shown as $E\left(\pi_{R}\right)\pm$ with additional effects.

(17) $$\begin{array}{l} U_{R}^{fr}=\frac{2\left(1+\lambda_{r}\right)}{2+\lambda_{r}}\pi_{R}-\frac{\lambda_{r}\left(1+\lambda_{r}\right)}{2+\lambda_{r}}\pi_{M}-\eta_{r}Var\left(\pi_{R}\right)\\ =\frac{2\left(1+\lambda_{r}\right)}{2+\lambda_{r}}\pi_{R}-\frac{\lambda_{r}\left(1+\lambda_{r}\right)}{2+\lambda_{r}}\pi_{M}-\eta_{r}\left[\left(p-w\right)\delta_{1}+\left(p_{m}-p_{r}\right)\delta_{2}\right] \end{array}$$

When $\lambda_{r}>0$, $\lambda_{m}=0$ and the manufacturer’s expected utility function is:

(18) $$\begin{array}{l} U_{M}=E\left(\pi_{M}\right)-\eta_{m}Var\left(\pi_{M}\right)=\\ \left(w-c_{m}\right)\left(\alpha -\beta p\right)+\left(c_{s}+\Delta -p_{m}\right)\left(a+bp_{r}\right)-\eta_{m}\left[\left(w-c_{m}\right)\delta_{1}+\left(c_{s}+\Delta -p_{m}\right)\delta_{2}\right] \end{array}$$

According to backwards induction, Equation (17) carries out the first-order derivation of p and $p_{r}$ respectively, $\begin{array}{l} \frac{\partial U_{R}{}^{fr}}{\partial p}=0,\frac{\partial U_{R}{}^{fr}}{\partial p_{r}}=0 \end{array}$, and we get:

(19) $$\begin{array}{l} \begin{array}{l} p^{fr}=\frac{\left(2+\lambda_{r}\right)\left(1+\lambda_{r}\right)\left(\alpha +\beta w\right)-\lambda_{r}\left(1+\lambda_{r}\right)\left(\alpha +\beta c_{m}\right)-\eta_{r}\delta_{1}\left(2+\lambda_{r}\right)}{4\beta \left(1+\lambda_{r}\right)}\\ p_{r}^{fr}=\frac{\left(2+\lambda_{r}\right)\left(1+\lambda_{r}\right)\left(p_{m}b-a\right)-\lambda_{r}\left(1+\lambda_{r}\right)\left[\left(c_{s}+\Delta \right)b-a\right]+\eta_{r}\delta_{2}\left(2+\lambda_{r}\right)}{4b\left(1+\lambda_{r}\right)} \end{array}\} \end{array}$$

When Equation (19) is put into Equation (18), we get the first order partial derivative of w and $p_{m}$, as 0. Then, with Equation (18) we get the optimal wholesale, recycle and transfer price of the manufacturer:

(20) $$\begin{array}{l} w^{fr^{*}}=\frac{2\alpha \left(1+\lambda_{r}\right)+2\beta c_{m}{\left(1+\lambda_{r}\right)}^{2}+\eta_{r}\delta_{1}\left(2+\lambda_{r}\right)-4\eta_{m}\delta_{1}\left(1+\lambda_{r}\right)}{2\beta \left(2+\lambda_{r}\right)\left(1+\lambda_{r}\right)}\\ p_{m}^{fr^{*}}=\frac{-2a\left(1+\lambda_{r}\right)+2b{\left(1+\lambda_{r}\right)}^{2}\left(c_{s}+\Delta \right)-\eta_{r}\delta_{2}\left(2+\lambda_{r}\right)+4\eta_{m}\delta_{2}\left(1+\lambda_{r}\right)}{2b\left(2+\lambda_{r}\right)\left(1+\lambda_{r}\right)} \end{array}\}$$

When Equation (20) is put into Equation (19) we get:

$$\begin{array}{l} p^{fr^{*}}=\frac{6\alpha \left(1+\lambda_{r}\right)+2\beta c_{m}\left(1+\lambda_{r}\right)-4\eta_{m}\delta_{1}\left(1+\lambda_{r}\right)-\eta_{r}\delta_{1}\left(2+\lambda_{r}\right)}{4\beta \left(1+\lambda_{r}\right)}\\ p_{r}^{fr^{*}}=\frac{-6a\left(1+\lambda_{r}\right)+2b\left(1+\lambda_{r}\right)\left(c_{s}+\Delta \right)+4\eta_{m}\delta_{2}\left(1+\lambda_{r}\right)+\eta_{r}\delta_{2}\left(2+\lambda_{r}\right)}{4b\left(1+\lambda_{r}\right)} \end{array}\}$$

The optimal sales volume is: $Q^{fr^{*}}=\frac{-2\alpha \left(1+\lambda_{r}\right)-2\beta c_{m}\left(1+\lambda_{r}\right)+4\eta_{m}\delta_{1}\left(1+\lambda_{r}\right)+\eta_{r}\delta_{1}\left(2+\lambda_{r}\right)}{4\left(1+\lambda_{r}\right)}$

The optimal recycle volume is: $G^{fr^{*}}=\frac{-2a\left(1+\lambda_{r}\right)+2b\left(c_{s}+\Delta \right)\left(1+\lambda_{r}\right)+\eta_{r}\delta_{2}\left(2+\lambda_{r}\right)+4\eta_{m}\delta_{2}\left(1+\lambda_{r}\right)}{4\left(1+\lambda_{r}\right)}$

**Proposition** **7.** *When the manufacturer shows risk aversion behaviors and the retailer shows both fairness concerns and risk aversion behaviors, the optimal wholesale price of the manufacturer increases with the increase in the retailer’s risk aversion coefficient*$\eta_{r}$*and the potential demand variance*$\delta_{1}$*, while the optimal wholesale price of the manufacturer decreases with the increase in the manufacture’s risk aversion coefficient*$\eta_{m}$*and potential demand variance*$\delta_{1}$*. When*$2\left(\beta c_{m}-\alpha \right){\left(1+\lambda_{r}\right)}^{2}-\eta_{r}\delta_{1}{\left(2+\lambda_{r}\right)}^{2}+4\eta_{m}\delta_{1}{\left(1+\lambda_{r}\right)}^{2}<0$*, the optimal wholesale price of the manufacturer decreases with the increase in the retailer’s fairness concern coefficient*$\lambda_{r}$.

**Proof**  $$\frac{\partial w}{\partial \eta_{r}\delta_{1}}=\frac{1}{2\beta \left(1+\lambda_{r}\right)}>0,\frac{\partial w}{\partial \eta_{m}\delta_{1}}=-\frac{2}{\beta \left(2+\lambda_{r}\right)}<0$$

where $2\left(\beta c_{m}-\alpha \right){\left(1+\lambda_{r}\right)}^{2}-\eta_{r}\delta_{1}{\left(2+\lambda_{r}\right)}^{2}+4\eta_{m}\delta_{1}{\left(1+\lambda_{r}\right)}^{2}<0$, $\frac{\partial w^{fr^{*}}}{\partial \lambda_{r}}<0$.

Proposition 7 shows that compared with the situation when both sides have risk aversion, the retailer’s fairness concerns do not change the trend of the manufacturer’s optimal wholesale price adjustment with the changes of the risk aversion coefficient for both sides. When retailers are clearly concerned with risk aversion, the wholesale price of the manufacturer will decrease as a result of the reduced bargaining power.

**Proposition** **8.** *When manufacturers have risk aversion, retailers have fairness concerns and risk aversion. The retailer’s optimal sales price increases with the increase in its fairness concern coefficient*$\lambda_{r}$*and decreases with increases in the retailer’s risk aversion coefficient*$\eta_{r}$*. The potential demand variance*$\delta_{1}$*decreases with the increases in manufacturer’s risk aversion coefficient*$\eta_{m}$*and the potential demand variance*$\delta_{1}$*. The retailer’s optimal recycle price decreases with the increase in its fairness concern coefficient*$\lambda_{r}$*and increases with the increases in its risk aversion coefficient*$\eta_{r}$*and the potential demand variance*$\delta_{2}$*. The increase in the manufacturer’s risk aversion coefficient*$\eta_{m}$*is the potential demand variance*$\delta_{2}$.

**Proof**  $$\frac{\partial p^{fr^{*}}}{\partial \lambda_{r}}=\frac{\eta_{r}\delta_{1}}{8\beta {\left(1+\lambda_{r}\right)}^{2}}>0$$

$$\frac{\partial p^{fr^{*}}}{\partial \eta_{m}\delta_{1}}=-\frac{1}{2\beta }<0,\;\frac{\partial p^{fr*}}{\partial \eta_{r}\delta_{1}}=-\frac{2+\lambda_{r}}{8\beta \left(1+\lambda \right)}<0$$

$$\frac{\partial p_{r}^{fr^{*}}}{\partial \lambda_{r}}=-\frac{\eta_{r}\delta_{2}}{8b{\left(1+\lambda_{r}\right)}^{2}}<0$$

$$\frac{\partial p_{r}^{fr^{*}}}{\partial \eta_{m}\delta_{2}}=\frac{2+\lambda_{r}}{2b\left(1+\lambda_{r}\right)}>0,\;\frac{\partial p_{r}^{fr^{*}}}{\partial \eta_{r}\delta_{2}}=\frac{2+\lambda_{r}}{8b\left(1+\lambda_{r}\right)}>0$$

Proposition 8 shows that compared with the situation when both sides have risk aversion, the retailer’s fairness concerns do not change the trend of the adjustment for the optimal wholesale price and recycle price with the changes of the risk aversion coefficient for both sides. The fact is, with increase in the retailer’s fairness concern coefficient, its bargaining power increases so retailers increase their proportion of profits in the supply chain by increasing sale prices and reducing recycle prices. That is why, when both fairness concerns and risk aversion exist, retailers should know which element’s influence is greater.

**Proposition** **9.** *(1)*$w^{fr^{*}}<w^{ra^{*}}$*(2)*$p^{fr^{*}}>p^{ra^{*}}$.

**Proof**  $$w^{fr^{*}}-w^{ra^{*}}=\frac{-\lambda_{r}\alpha \left(1+\lambda_{r}\right)+\lambda_{r}\beta c_{m}\left(1+\lambda_{r}\right)-\lambda_{r}\eta_{r}\delta_{1}\left(2+\lambda_{r}\right)+2\lambda_{r}\eta_{m}\delta_{1}\left(1+\lambda_{r}\right)}{2\beta \left(2+\lambda_{r}\right)\left(1+\lambda_{r}\right)}<0,\;w^{fr^{*}}<w^{ra^{*}}$$

$$p^{fr^{*}}-p^{ra^{*}}=\frac{3\alpha \left(1+\lambda_{r}\right)+\beta c_{m}\left(1+\lambda_{r}\right)-2\eta_{m}\delta_{1}\left(1+\lambda_{r}\right)-\eta_{r}\delta_{1}}{8\beta \left(1+\lambda_{r}\right)}>0,p^{fr^{*}}>p^{ra^{*}}$$

Proposition 9 shows that fairness concerns’ effect on the optimal price strategy for manufacturers and retailers is more direct than risk aversion. The optimal wholesale price of the manufacturer is inversely proportional to the retailer’s fairness concern coefficient s, so the optimal wholesale price will be lower than when both sides have risk aversion. The optimal sales price of the retailer is directly proportional to the retailer’s fairness concern coefficient s, so the optimal sales price is higher than when both sides have risk aversion.

**Proposition** **10.** *For both manufacturers and retailers, either fairness concern or risk aversion is concerned. For new products, the wholesale price is the most noticeably affected, followed by the sales price, while for the used products, the transfer price is the most noticeably affected, followed by the recycle price.*

**Proof**  (3) is the optimal reaction function for retailers when the wholesale price and the recycle price are given. $\frac{\partial p}{\partial w}=\frac{1}{2}$, $\frac{\partial p_{r}}{\partial p_{m}}=\frac{1}{2}$ is based on the derivation method of the composition function:

$$\frac{\partial p}{\partial \lambda }=\frac{\partial p}{\partial w}\frac{\partial w}{\partial \lambda }=\frac{1}{2}\frac{\partial w}{\partial \lambda }<\frac{\partial w}{\partial \lambda }$$

$$\frac{\partial p}{\partial \eta \delta }=\frac{\partial p}{\partial w}\frac{\partial w}{\partial \eta \delta }=\frac{1}{2}\frac{\partial w}{\partial \eta \delta }<\frac{\partial w}{\partial \eta \delta }$$

$$\frac{\partial p_{r}}{\partial \lambda }=\frac{\partial p_{r}}{\partial p_{m}}\frac{\partial p_{m}}{\partial \lambda }=\frac{1}{2}\frac{\partial p_{m}}{\partial \lambda }<\frac{\partial p_{m}}{\partial \lambda }$$

$$\frac{\partial p_{r}}{\partial \eta \delta }=\frac{\partial p_{r}}{\partial p_{m}}\frac{\partial p_{m}}{\partial \eta \delta }=\frac{1}{2}\frac{\partial p_{m}}{\partial \eta \delta }<\frac{\partial p_{m}}{\partial \eta \delta }$$

## 7. Data Simulation

To better explain the application of the model, we will test the utility model under the influence of the factors mentioned above with numerical simulation to show how fairness concerns and risk aversion can affect the optimal price and profit in a closed-loop supply chain.

Suppose the product demand function is D(p)=100−5p, the function of the recycle volume is $G\left(p_{r}\right)=10+20p_{r}$, the unit production cost of new products is $c_{m}=10$, the cost of remanufacturing $c_{r}=6$, the government subsidy is $c_{s}=1$. λ and η belongs to [0,1]. Utilize the situation when both sides are risk-neutral and fair-neutral as reference.

**Figure 2:**
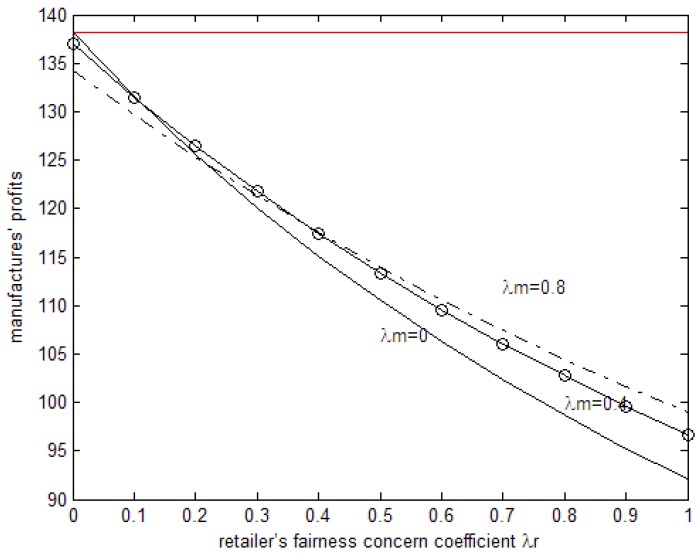
The effects of the fairness concern coefficient on the manufacturer’s profits.

The horizontal axis in Figure 2 is the manufacturer’s profit when it is fair-neutral. As can be seen in Figure 1, when both sides have fairness concerns the profit of the manufacturer decreases with the increase in the retailer’s fairness concern coefficient. When the fairness concern coefficient is relatively low, the profit of the manufacturer decreases, while its own fairness concern coefficient increases. When the fairness concern coefficient becomes higher, the profit of the manufacturer increases with the increase in its own fairness concern coefficient. When both sides have fairness concerns, the manufacturer’s profit is lower than when fair-neutral in the case of the retailer’s fairness concern coefficient being(>0) and the manufacturer’s fairness concern coefficient being(<1). When the manufacturer’s fairness concern coefficient is 0, that is to say, only when retailers have fairness concerns, the manufacturer’s profit will be lower than that of when they are fair-neutral but higher than that of when both sides have fairness concerns.

**Figure 3:**
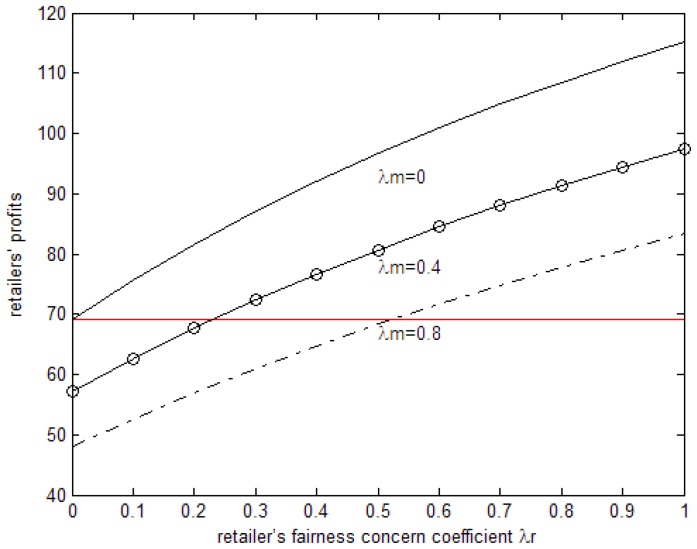
The effects of the fairness concern coefficient on the retailer’s profits.

The line parallel to the horizontal axis in Figure 3 is the retailer’s profit when it is fair-neutral. From Figure 2 we can see that, under the parameter above, when both sides have fairness concerns, the retailer’s profit increases with increases in the retailer’s fairness concern coefficient while it decreases with the increase in the manufacturer’s fairness concern coefficient. When the retailer’s fairness concern coefficient is relatively low, the profit of the retailer is lower than that of when it is fair-neutral but, with the increase in the retailer’s fairness concern coefficient, the profit is higher. When the manufacturer’s fairness concern coefficient is relatively low, the profit of the retailer is higher than that of when it is fair-neutral but, with the increase in the manufacturer’s fairness concern coefficient, the profit is lower. When the manufacturer’s fairness concern coefficient is 0, that is to say, only when retailers have fairness concerns, the manufacturer’s profit is higher than when they are fair-neutral and higher than when both sides have fairness concerns.

**Figure 4:**
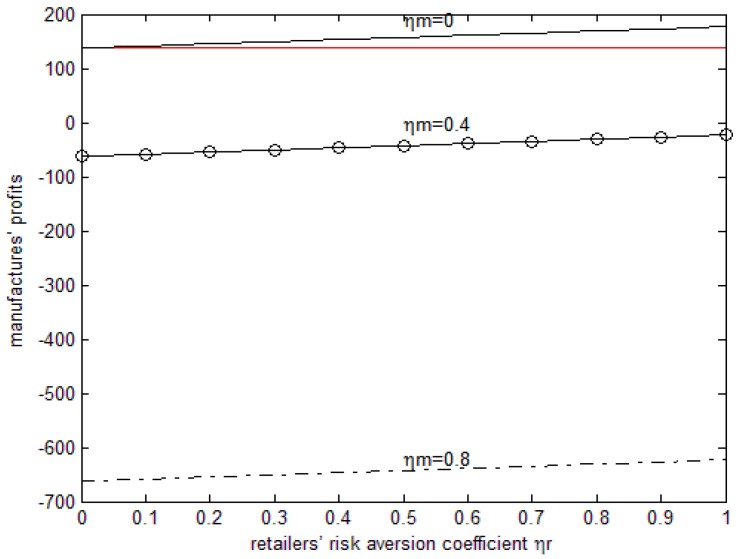
The effects of the risk aversion coefficient on the manufacturer’s profits.

The line parallel to the horizontal axis in Figure 4 is the manufacturer’s profit when they are risk-neutral. As can be seen in Figure 3 under the parameter above, when both sides have risk aversion the profit of the manufacturer increases with the increase in the retailer’s risk aversion coefficient, while it decreases with the increase in the manufacturer’s risk aversion coefficient. However high both sides’ fairness concern coefficient is (<1), the manufacturer’s profit will be lower than when they are risk-neutral. When the manufacturer’s fairness concern coefficient is 0, that is to say, only when retailers have fairness concerns, the manufacturer’s profit is higher than when they are risk-neutral and when both sides have risk concerns.

**Figure 5:**
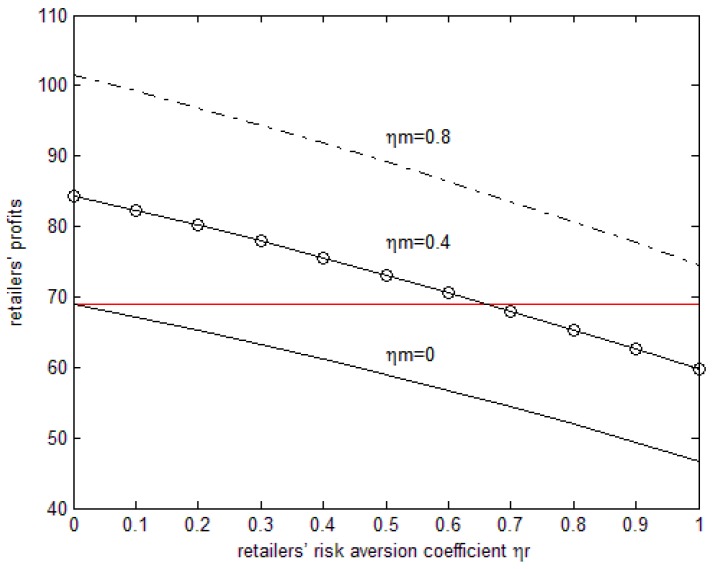
The effects of the risk aversion coefficient on the retailer’s profits.

The line parallel to the horizontal axis in Figure 5 is the retailer’s profit when they are risk-neutral. From Figure 4 we can see that under the parameters above, when both sides have risk aversion the retailer’s profit decreases with the increase in retailer’s risk aversion coefficient, while there are increases in the manufacturer’s risk aversion coefficient. When the retailer’s risk aversion coefficient is relatively low, the profit of the retailer is higher than that of risk-neutral but, with the increase in the retailer’s risk aversion coefficient, the profit is lower. When the manufacturer’s risk aversion coefficient is relatively low, the profit of the retailer is lower than that of risk-neutral but, with the increase in the manufacturer’s risk aversion coefficient, the profit is higher. When the manufacturer’s risk aversion coefficient is 0, that is to say, only when retailers have risk aversion, the manufacturer’s profit will be lower than when they are risk-neutral and when both sides have risk aversion.

**Figure 6:**
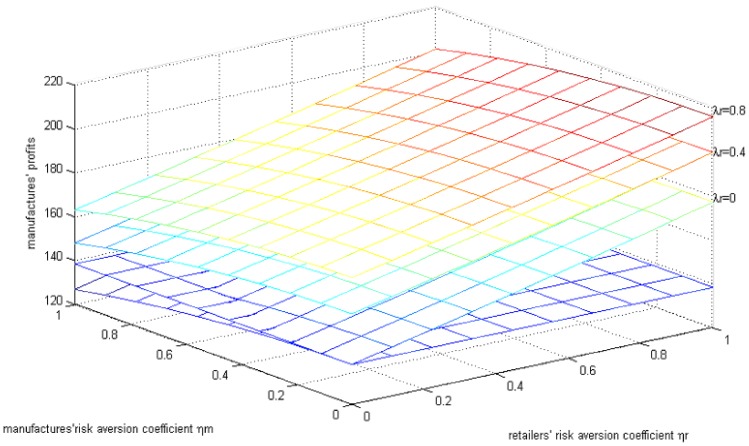
The effects of the risk aversion coefficient and fairness concern coefficient on the manufacturer’s profits.

The line parallel to the horizontal axis in Figure 6 is the manufacturer’s profit when it is fair-neutral and risk-neutral. As can be seen from Figure 5 under the parameters above, when the manufacturer has risk aversion and the retailer has both risk aversion and fairness concerns, the profits of the manufacturer increases with the increase in the retailer’s risk aversion coefficient, decreases with the increase in the manufacturer’s risk aversion coefficient, and increases with the increase in the retailer’s coefficient of equity concern. That is to say, the change of the retailer’s coefficient of equity concern positively affects the manufacturer’s profits. For manufacturers, the profits are higher when the retailer’s risk coefficient of aversion and the fairness concern coefficient s are higher and their own risk aversion coefficient is lower.

**Figure 7:**
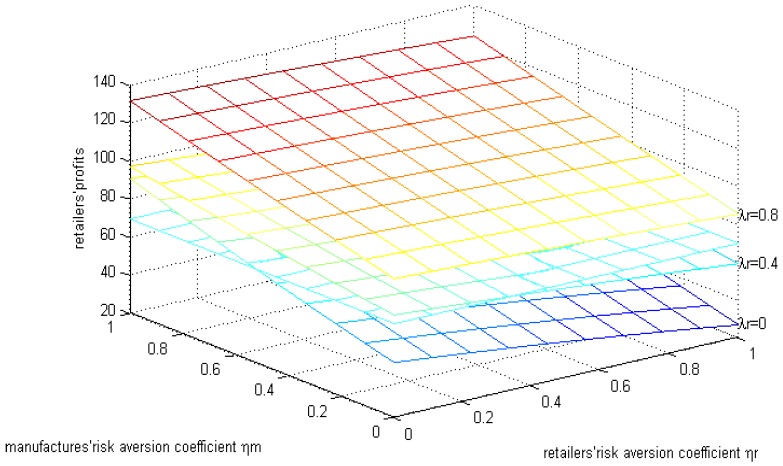
The effects of the risk aversion coefficient and fairness concern coefficient on the retailer’s profits.

The line parallel to the horizontal axis in Figure 7 is the retailer’s profit when it is fair-neutral and risk-neutral. As can be seen in Figure 6 under the parameters above, when the manufacturer has risk aversion and the retailer has risk aversion and fairness concerns, the retailer’s profit decreases with the increase in the retailer’s risk aversion coefficient, and increases with the increase in the retailer’s fairness concern coefficient. For retailers, their profit will be higher when the manufacturer’s risk coefficient of aversion and their own fairness concern coefficient are higher and their own risk aversion coefficient is lower.

## 8. Conclusions

In this paper, fairness concerns and risk aversion are applied to the analysis of the price strategy of a closed-loop supply chain. Three situations are discussed: (1) both manufacturers and retailers have fairness concerns, (2) both manufacturers and retailers have risk aversion, and (3)manufacturer have risk aversion and retailers have both risk aversion and fairness concerns. The simulation results show that risk aversion and fairness concerns can change the price strategy of wholesale price, transfer price, sale price and recycle price, and affect the expected profits of retailers and manufacturers. Specifically, when both sides have fairness concerns, the optimal sale price in the supply chain is highest. When both sides have risk aversion, the optimal recycle price in the supply chain is highest. 

The paper also shows that the irrational factors’ effects vary depending on whether they relate to the wholesale price, the sale price of new products, or the recycle and transfer price of used products. The wholesale price of new products is most affected, followed by the sale price. The transfer price is the most affected, followed by the recycle price which is consistent with the conclusions of other studies [30,31]. However, when the manufacturer has risk aversion and the retailer has both risk aversion and fairness concerns, the retailer’s fairness concerns have positive effects on both sides. For the simplicity of the model, this thesis mainly discussed two irrational factors. For further research, more factors can be included to further analyze how they affect the price strategy and various recycle channels.

Academically, the paper contributes to the literature by: (1) proposing a model to explore how manufacturers and retailers to price when considering fairness concerns and risk aversion in a closed-loop supply chain; (2)demonstrating the results that two irrational factors, fairness concerns and risk aversion, play an important role in the pricing decisions of closed-loop supply chains; and (3) demonstrating that the effects of irrational factors vary when they play a role in the different stages of pricing in a closed-loop supply chain.

Practically, the analytical model introduced offers anatomy in the sustainable operation of a closed-loop supply chain. The sustainable operation of a supply chain depends on continuous and positive interactions (i.e., action–response) among member organizations (e.g., manufacturers and retailers). By incorporating the two irrational factors into existing literature, practitioners now can make more comprehensive considerations when setting up a pricing strategy (a critical kind of “action”) that may affect responses. In such way, the continuity or discontinuity of inter-organizational interactions would in turn determine the sustainability or corruption of a supply chain system.

There are several recommendations for future research. First, studies can be carried out with consideration of the coordinated pricing of lower closed-loop supply chains and their application in the selection of different recycling channels. Second, as we only examined two irrational factors in our model, future studies could incorporate more factors in addition to risk aversion and fairness concerns. Third, as our model assumes independent decision making between manufacturers and retailers, future studies could incorporate the interdependence between decision makers in different parties to achieve a more complex and comprehensive explanation of the phenomenon.
